# Eighteen Years of Complication-Free Survival Following Inadvertent Left Ventricular Implantable Cardioverter Defibrillator (ICD) Lead Placement via Atrial Septal Defect: A Case Report

**DOI:** 10.7759/cureus.117698

**Published:** 2026-10-09

**Authors:** Omar Elboraey, Shehab Hasan, Muhammad Farooq, Ian Schofield, Irfan M Ahmed

**Affiliations:** 1 Cardiology Department, East Cheshire National Health Service (NHS) Trust, Macclesfield, GBR; 2 Emergency Department, Lancashire Teaching Hospitals National Health Service (NHS) Trust, Preston, GBR; 3 Cardiology Department, Lancashire Teaching Hospitals National Health Service (NHS) Trust, Preston, GBR

**Keywords:** atrial septal defect (asd), congenital heart disease (chd), implantable cardioverter-defibrillator (icd), implantable electronic devices (cieds), inadvertent lead malposition in the left heart (ilmlh)

## Abstract

Implantable cardioverter defibrillators (ICDs) are recommended for primary or secondary prevention in multiple cardiac conditions. One of the recognised complications during implantation of cardiac implantable electronic device (CIED) is inadvertent lead malposition in the left heart (ILMLH), which can lead to further thromboembolism and infective endocarditis.

In this paper, we present a unique case of a patient who had an accidental ICD lead implanted into the left ventricle via an atrial septal defect (ASD). Evidence on the management of such a complication is rare. A watchful-wait approach with appropriate anticoagulation was adopted here, resulting in an uneventful 18-year survival. We also navigate through the current evidence on the recognition and management of such cases.

## Introduction

Device therapy with an implantable cardioverter-defibrillator (ICD) is recommended to reduce mortality in multiple cardiac conditions for primary or secondary prevention. In the European Society of Cardiology (ESC) Heart Failure guidelines 2021 and its focused update in 2023, ICD therapy was recommended for patients with Heart Failure with reduced Ejection Fraction (HFrEF) less than 35% who remain symptomatic with New York Heart Association (NYHA) class II-III despite Optimal Medical Therapy (OMT) [1]. Before that, the Sudden Cardiac Death in Heart Failure Trial (SCD-HeFT) demonstrated the benefit of ICD therapy in reducing mortality in patients with HFrEF [2].

ICDs can be transvenous, subcutaneous or extravascular systems. Transvenous systems include: single-lead devices in which the defibrillator coil is implanted in the right ventricle (RV), dual-coil devices with an additional defibrillator coil in the superior vena cava (SVC), dual-lead devices with an additional sensing/pacing lead in the right atrium (RA), and cardiac resynchronisation therapy devices with a defibrillator (CRT-D), which has an extra pacing lead in the coronary sinus. Mispositioning of the ICD coil lead in the left ventricle (LV) is rare and carries several potential complications, such as thromboembolism, mitral valve injury, and infective endocarditis. It can occur via inadvertent arterial access, patent foramen ovale (PFO), atrial septal defect (ASD) traversal, or perforation of an interatrial or interventricular septum [3].

It is usually diagnosed using imaging modalities such as chest X-ray (CXR), chest computed tomography (CT), or echocardiography. Management options for malpositioned leads in the LV include extraction or long-term anticoagulation [4].

In this case, we present an interesting case of a patient who had an inadvertently implanted ICD in the LV and survived without complications for 18 years. We review the current literature, relevant similar cases, and extract lessons.

## Case presentation

A 72-year-old gentleman presented in 2007 with syncope secondary to non-sustained ventricular tachycardia (NSVT). He had a background of severe ischaemic left ventricular systolic dysfunction (LVSD) with ejection fraction <35%, and a large ASD with a left-to-right shunt. As he was asymptomatic from the ASD, when reviewed by cardiothoracic surgeons, surgical closure was not recommended. Since the patient was asymptomatic with ASD, a shared decision between the patient and surgeon was reached in 2005 not to operate on it. In view of the presence of an intracardiac shunt, he was started on regular warfarin as prophylaxis for paradoxical embolism and stroke before the novel oral anticoagulants (NOACs) were licensed for this purpose.

He was referred for electrophysiology (EP) studies and ventricular tachycardia (VT) ablation. Unfortunately, the procedure was not successful due to VT non-inducibility. Hence, in view of the severe LVSD and history of NSVT, he was eligible for ICD implantation for secondary prevention according to the SCD-HeFT trial criteria. A dual-coil ICD was implanted with initially satisfactory sensing (R wave >7) and pacing (<1 mV) thresholds. However, intra-operative testing with 17J and 21J failed to terminate VT/VF (ventricular fibrillation) and external cardioversion was required. Therefore, another device with a higher output (36J) was implanted a week later.

Four years later, on a regular device check, battery life was found to be significantly reduced, and a pulse generator change procedure was planned. In the pre-procedure assessment, transthoracic echocardiogram showed that the RV lead was coursing from the right atrium to the left atrium via the ASD, then across the mitral valve to the left ventricle (Figures 1-5).

**Figure 1:**
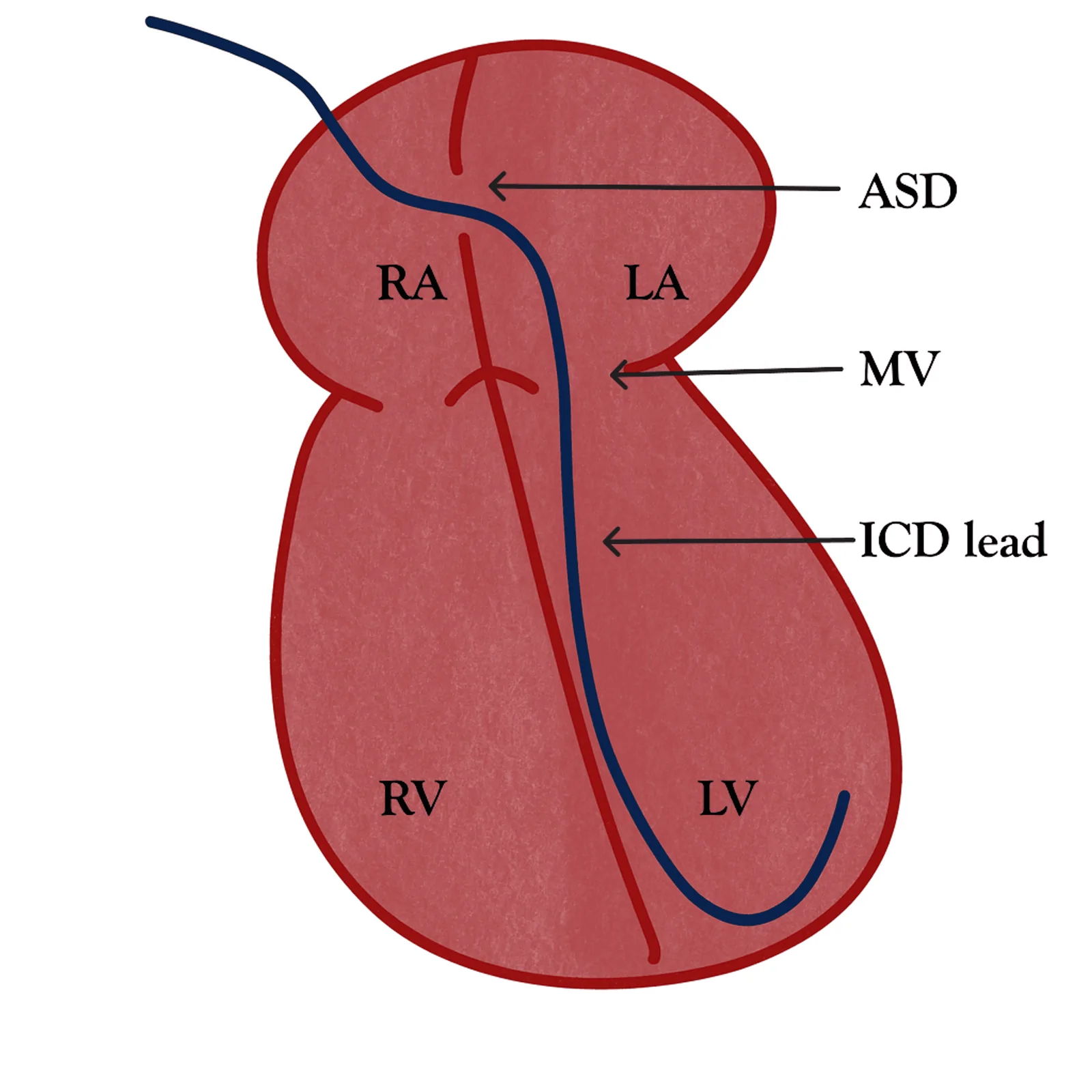
Diagram showing the ICD lead course during implantation RA, Right Atrium; RV, Right Ventricle; LA, Left Atrium; LV, Left Ventricle; ASD, Atrial Septal Defect; MV, Mitral Valve; ICD, Implantable Cardioverter Defibrillator. The unusual course of the ICD lead crossing from the RA to the LA through the ASD, then to the LV instead of the expected course from RA to RV. Image credit: Dr. Omar Elboraey, using the digital painting tool Procreate app for iPad (Savage Interactive Pty Ltd., Hobart, Tasmania, Australia).

**Figure 2:**
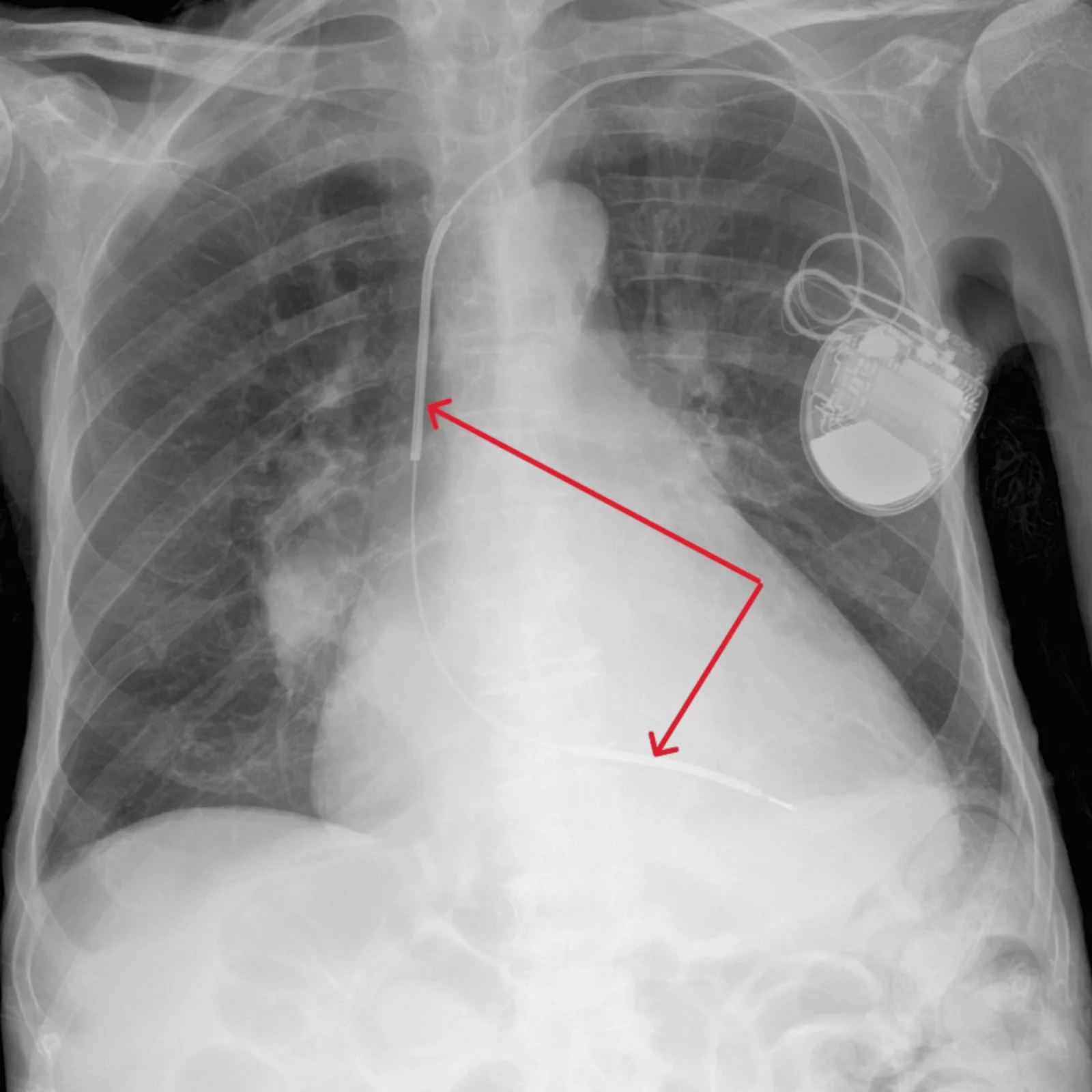
Chest X-ray showing the dual coil ICD course Red arrows pointing towards the lead coils. The top one lies in the IVC and the bottom one in the LV. ICD, Implantable cardioverter defibrillator; IVC: inferior vena cava; LV: left ventricle.

**Figure 3:**
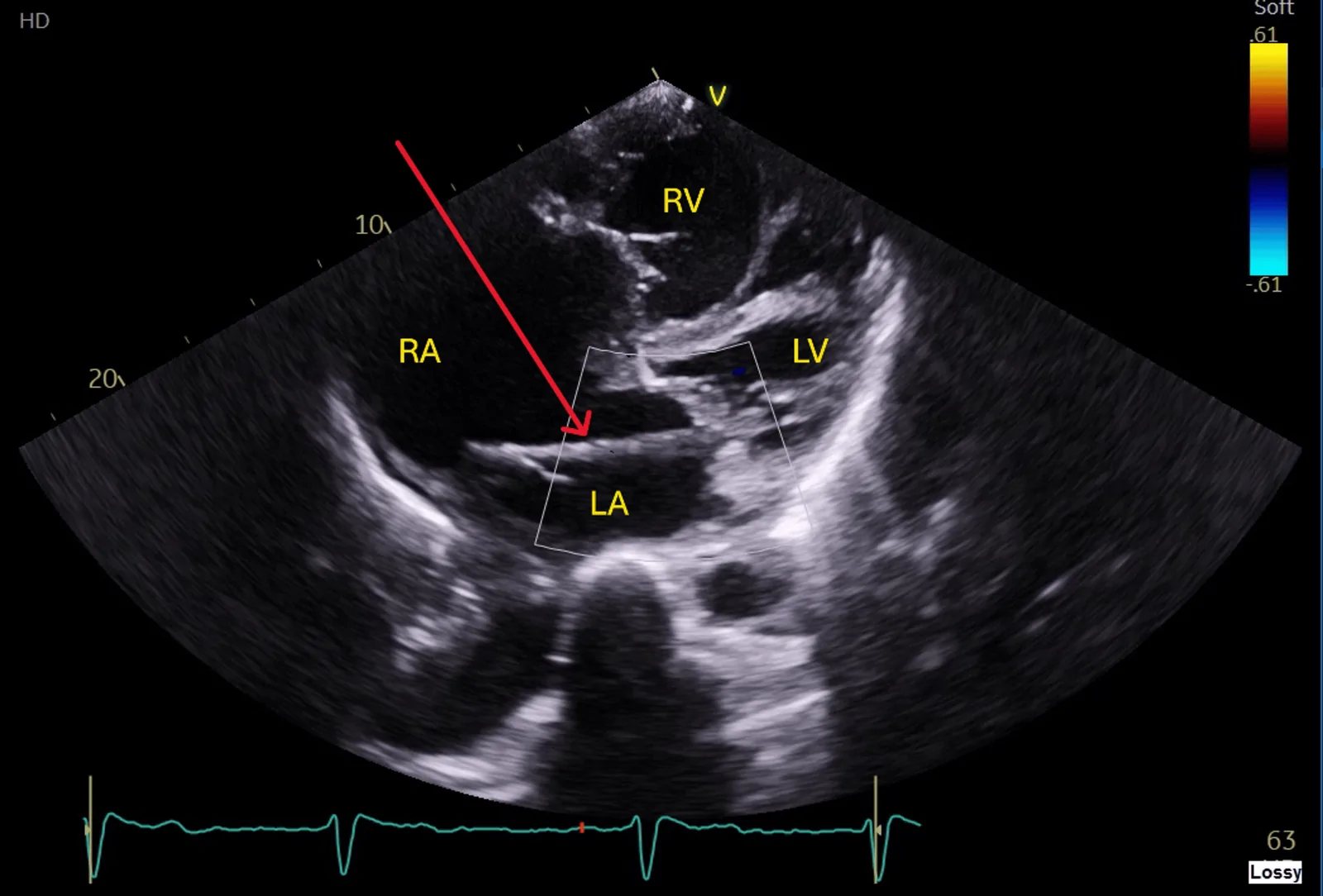
Transthoracic echocardiogram, apical four chambers view showing ICD lead crossing from RA to LA through ASD ICD, Implantable cardioverter defibrillator; RA: right auricle; LA: left auricle; ASD: atrical septal defect.

**Figure 4:**
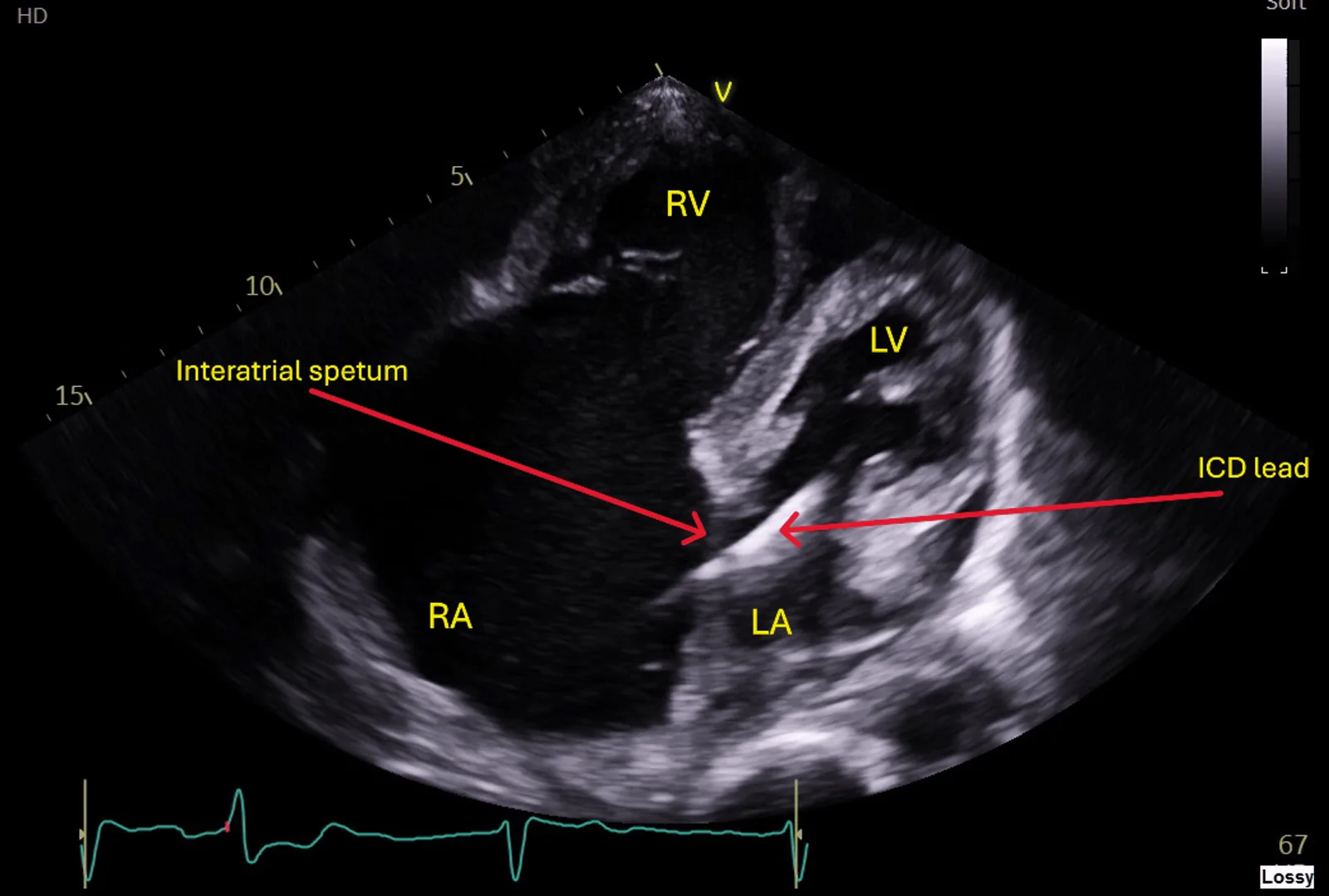
Transthoracic echocardiogram, apical four champers view showing ICD lead crossing from LA to LV through the mitral valve ICD, Implantable cardioverter defibrillator; RA: right auricle; LA: left auricle.

**Figure 5:**
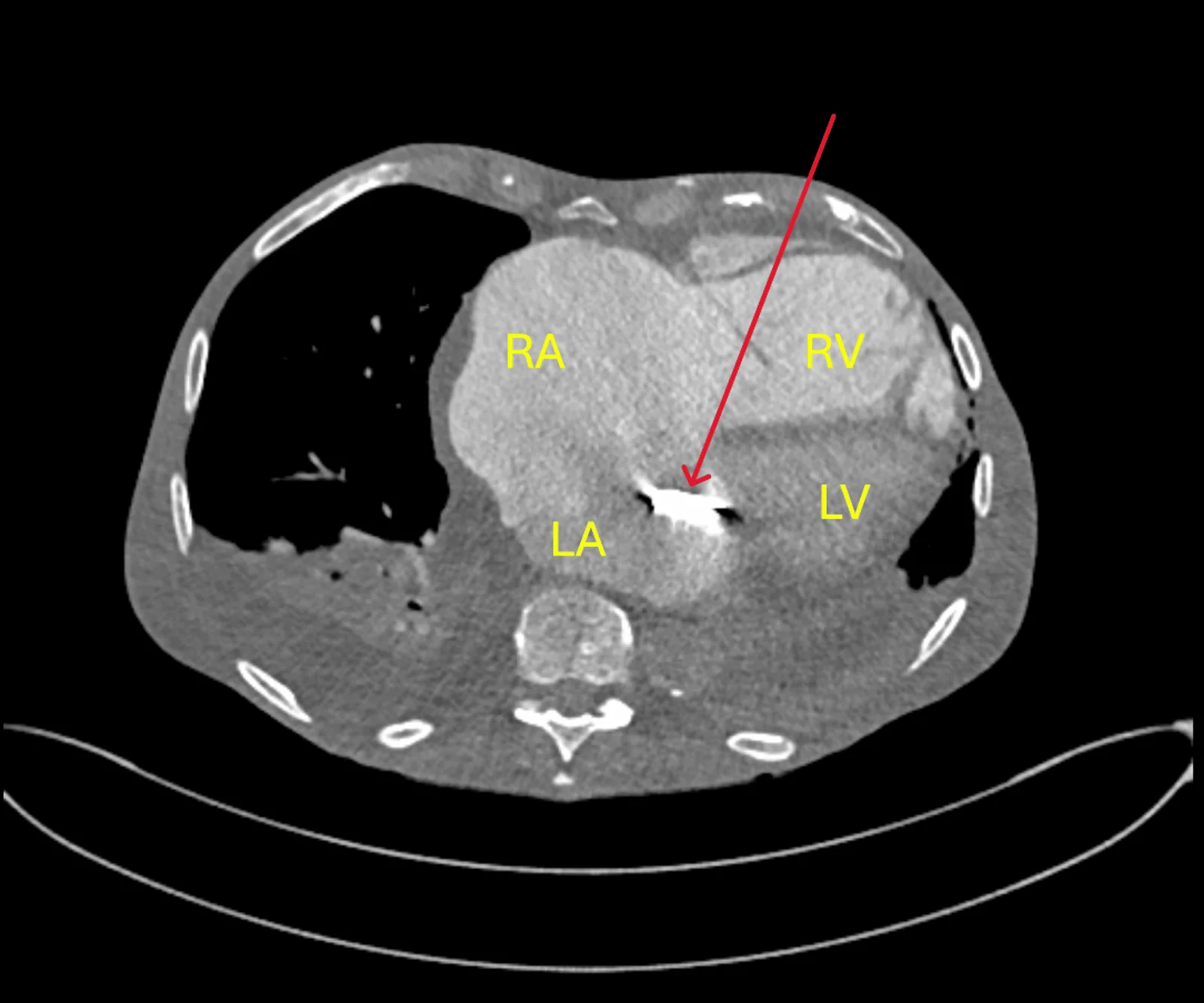
Thorax CT showing IVD lead course from RA to LA then LV ICD, Implantable cardioverter defibrillator; RA: right auricle; LA: left auricle; LV: left ventricle.

Since the lead has been in the LV for four years with expectations of fibrosis around it, the patient is already on anticoagulation and in the absence of systemic embolic events, device infection, or further deterioration of LV function, the decision was made to leave the ICD lead in situ and continue warfarin for anticoagulation, aiming for a target international normalised ratio (INR) of 2-3 rather than extracting the lead. In 2015, anticoagulation with novel oral anticoagulants (NOAC) was considered but not used as these drugs were not licensed for this purpose.

During the course of the patient's management, he was commenced on amiodarone, but was later stopped due to thyrotoxicosis, and it was switched to sotalol.

Despite the unusual LV lead position, the patient remained clinically stable with no thromboembolism, infective endocarditis or device-related complications over 18 years of close follow-up.

## Discussion

In the latest ESC Heart Failure guidelines 2021 and its focused update 2023, ICD therapy was recommended class I for secondary prevention to reduce the risk of sudden death and all-cause mortality in patients who recovered from ventricular arrhythmias causing haemodynamic instability and who are expected to survive more than one year with good functional status. It was also recommended for primary prevention in patients with heart failure with reduced ejection fraction (HFrEF) less than 35% due to an ischaemic aetiology who remain symptomatic, New York Heart Association (NYHA) class II-III, despite more than three months of optimal medical therapy, as class I and as class IIa in patients with non-ischaemic aetiology [1].

The SCD-HeFT trial, published in 2005, was one of the early trials to prove the benefit of ICD therapy in reducing overall mortality in congestive heart failure patients with NYHA class II or III due to ischaemic and non-ischaemic aetiologies [2].

Currently available ICD devices are transvenous, subcutaneous or extra-vascular. According to the ESC guidelines, transvenous pacing is preferred when indicated. ICDs can be single- or dual-chamber, with leads usually placed in the right ventricle±the right atrium. Inadvertent placement of the lead into the left ventricle is one complication that may arise during the procedure. This can be through inadvertent arterial access by puncturing the subclavian artery coursing retrogradely to the aorta, then the left ventricle. Through venous access, it can pass to the left atrium through an ASD, PFO or perforation of the interatrial septum. Furthermore, it can occur by coursing from the RV to the LV through a ventricular septal defect (VSD) or a perforation of the interventricular septum.

During the procedure, fluoroscopic guidance is used to place the ICD lead in the RV and, in dual-coil devices, in the RA. Images are obtained from multiple projections: posterior-anterior (PA), left anterior oblique (LAO) and right anterior oblique (RAO) to ensure correct placement in the RV.

After initial placement in PA view, the lead should point towards the observer in the RAO view, not anteriorly towards the anterior RV wall, to avoid perforation. In the LAO view, the lead should cross the tricuspid valve plane and be fully inserted in the RV. Post-procedure, CXRs in PA and lateral views are obtained to confirm correct lead positioning and exclude complications such as pneumothorax or cardiac perforation.

Lead mispositioning in the left heart can still occur at a rate of 0.07%-0.34% [3,4]. The first case was reported in 1969 [5]. A recent systematic review suggested that this complication is often unrecognised or under-reported [3]. This is usually diagnosed on transthoracic echocardiography, CXR, CT or 12-lead ECG showing right bundle branch block (RBBB) morphology in pacemakers [6].

Left-sided endocardial leads have a risk of thrombus formation, leading consequently to systemic embolisation, including ischemic stroke or transient ischemic attack (TIA), and they can also cause damage to left-sided heart valves. Current guidelines do not provide specific recommendations for managing such cases. Still, options include lead extraction via percutaneous or surgical procedures and lifelong anticoagulation if extraction was not attempted or was unsuccessful [4,7]. In this case, warfarin was used to achieve an INR >2.5 for thromboembolic prophylaxis. Antiplatelet therapy doesn’t provide enough protection from thromboembolic risk, and NOACs are not licensed for this purpose [8-11].

This case is unique for long-term complication-free survival for 18 years despite inadvertent LV placement of the ICD lead, discovered four years after the procedure. It highlights the value of adequate anticoagulation with warfarin to prevent thromboembolic complications when lead extraction is not undertaken. Finally, it underscores the importance of using multiple fluoroscopic projections during lead placement for cardiac implantable electronic device (CIED) implantation, particularly in patients with congenital heart disease.

## Conclusions

This case demonstrates an exemplary, conservatively well-managed patient with an inadvertently implanted ICD lead in the LV once discovered. Although it is potentially hazardous with multiple possible complications, this patient survived with no device-related complications through careful monitoring and lifelong anticoagulation. It also highlights the need for imaging guidance across multiple projections during and after the procedure to ensure correct lead position, particularly in patients with known congenital heart defects. This patient’s complication-free survival for 18 years provides valuable insight into long-term outcomes in such a scenario. We also acknowledge the study's limitations, such as the lack of periprocedural details and imaging studies, which would have added further value. This was mainly due to the long period between the procedure and the report, as well as the split of the patient's care among multiple centres, which made it challenging to collect relevant data.
